# Supplementary material for: New methods for quantifying rapidity of action potential onset differentiate neuron types
Source: PLoS One. 2021 Apr 8;16(4):e0247242. doi: 10.1371/journal.pone.0247242 (PMC8032118; doi:10.1371/journal.pone.0247242)
Supplement: S1 Fig — The first 9 neurons are deep pyramidal neurons, while the following 8 neurons are superficial pyramidal neurons. Blue circle: Values using spline interpolation. Red diamond: Values using pchip interpolation. (DOCX) [file pone.0247242.s001.docx]

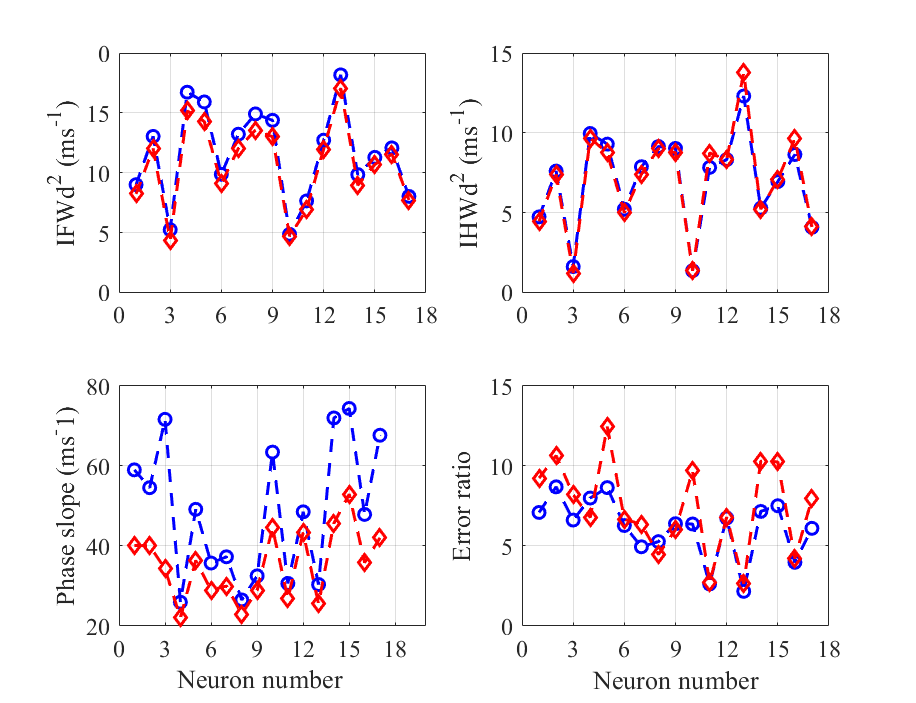


**S1 Fig. Pooled mean values for hippocampal pyramidal neuron rapidity.** The first 9 neurons are deep pyramidal neurons, while the following 8 neurons are superficial pyramidal neurons. Blue circle: values using spline interpolation. Red diamond: values using pchip interpolation.
