# Supplementary material for: New methods for quantifying rapidity of action potential onset differentiate neuron types
Source: PLoS One. 2021 Apr 8;16(4):e0247242. doi: 10.1371/journal.pone.0247242 (PMC8032118; doi:10.1371/journal.pone.0247242)
Supplement: S3 Fig — Top left: The error-ratio value when the upper limit was set at varying voltages above onset potential and the lower limit was 9 ms before onset. Top right: The error-ratio value when the upper limit was set at varying percentages of the maximum V˙m and the lower limit was 9 ms before onset. Bottom left: The error-ratio value when the upper limit was set at varying absolute voltages above onset potential and the lower limit was 5 ms before onset. Bottom right: The error-ratio value when the upper limit was set at varying percentages of the maximum V˙ and the lower limit was 5 ms before onset. Blue diamonds represent the error ratio for the neuron labeled AL 133, and orange circles represent the error ratio for the neuron labeled AL 215 [19]. (DOCX) [file pone.0247242.s003.docx]

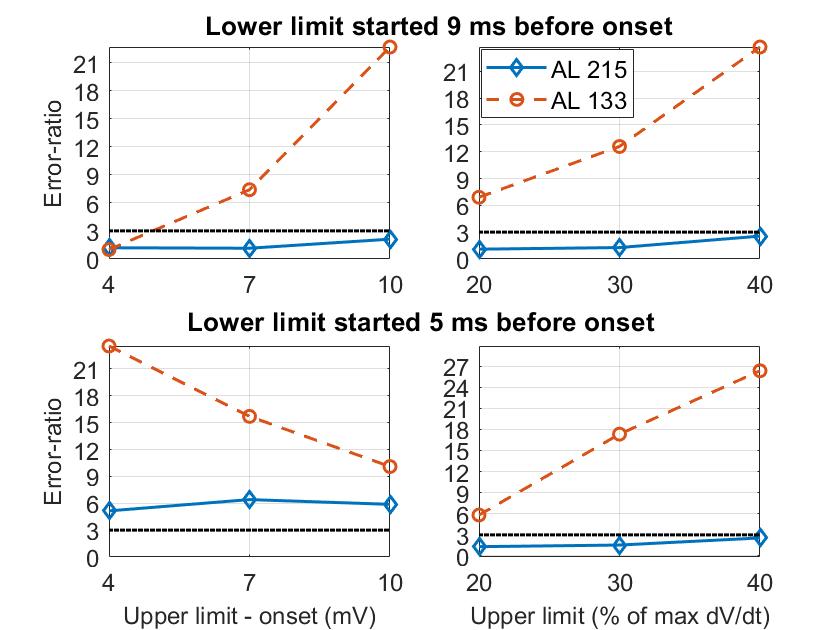


**S3 Fig.** **The effect on the error ratio of changing the upper and lower limits of data selection is compared for two neurons.** Top left: the error-ratio value when the upper limit was set at varying voltages above onset potential and the lower limit was 9 ms before onset. Top right: the error-ratio value when the upper limit was set at varying percentages of the maximum $\dot{V}_{m}$ and the lower limit was 9 ms before onset. Bottom left: the error-ratio value when the upper limit was set at varying absolute voltages above onset potential and the lower limit was 5 ms before onset. Bottom right: the error-ratio value when the upper limit was set at varying percentages of the maximum $\dot{V}$ and the lower limit was 5 ms before onset. Blue diamonds represent the error ratio for the neuron labeled AL 133, and orange circles represent the error ratio for the neuron labeled AL 215 [19].
