# Supplementary material for: New methods for quantifying rapidity of action potential onset differentiate neuron types
Source: PLoS One. 2021 Apr 8;16(4):e0247242. doi: 10.1371/journal.pone.0247242 (PMC8032118; doi:10.1371/journal.pone.0247242)
Supplement: S4 Fig — Black circles represent the V¨m points calculated from the raw recordings before applying any interpolation functions. The dotted red line shows the V¨m trace after applying the quadratic regression interpolation function (pchip), while the dashed blue line shows the V¨m trace after applying the cubic spline interpolation function (spline). (DOCX) [file pone.0247242.s004.docx]

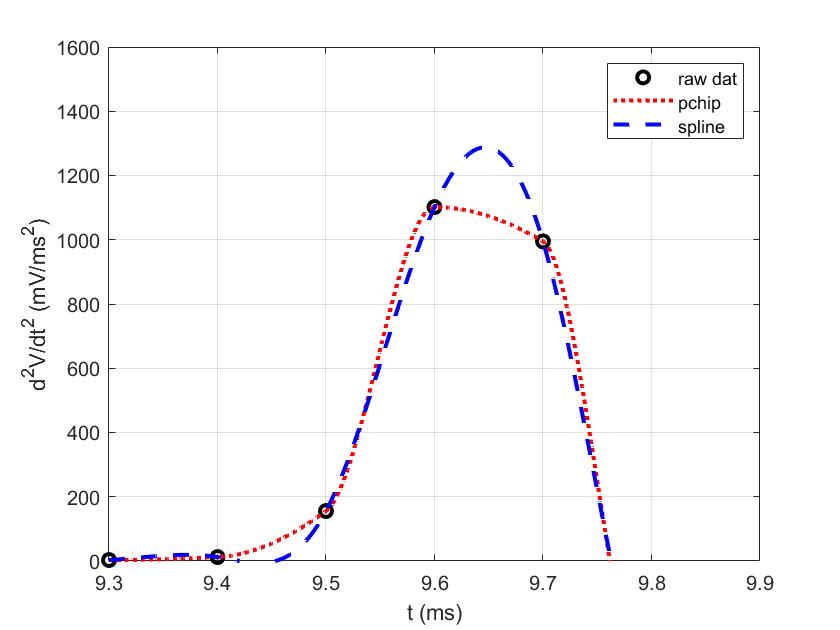


**S4 Fig. The impact of interpolation function on the shape and thus height of the** ${\ddot{\boldsymbol{V}}}_{\boldsymbol{m}}$ **peak**. Black circles represent the $\ddot{V}_{m}$ points calculated from the raw recordings before applying any interpolation functions. The dotted red line shows the $\ddot{V}_{m}$ trace after applying the quadratic regression interpolation function (pchip), while the dashed blue line shows the $\ddot{V}_{m}$ trace after applying the cubic spline interpolation function (spline)
