# Supplementary material for: New methods for quantifying rapidity of action potential onset differentiate neuron types
Source: PLoS One. 2021 Apr 8;16(4):e0247242. doi: 10.1371/journal.pone.0247242 (PMC8032118; doi:10.1371/journal.pone.0247242)
Supplement: S5 Fig — Blue circles show the mean phase slope value at different criterion levels for the hippocampal RS pyramidal neurons. Red squares show the mean phase slope value at different criterion levels for the hippocampal FS PVBCs. All APs that have maximum V˙m less than 45 mV/ms were excluded. Note that the rapidity for RS and FS hippocampal neurons cross using the phase slope method. (DOCX) [file pone.0247242.s005.docx]

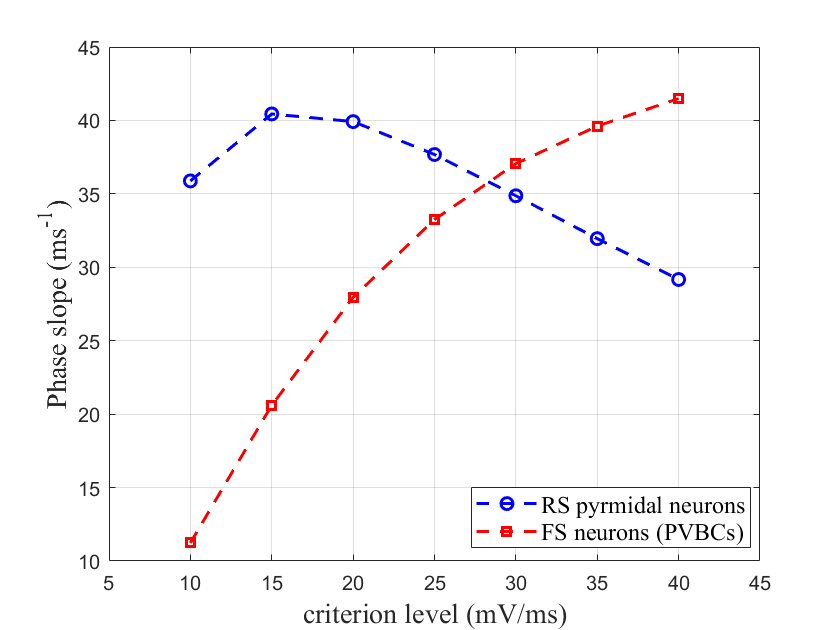


**S5 Fig. The impact of the onset criterion level on the phase slope for hippocampal neurons.** Blue circles show the mean phase slope value at different criterion levels for the hippocampal RS pyramidal neurons. Red squares show the mean phase slope value at different criterion levels for the hippocampal FS PVBCs. All APs that have maximum $\dot{V}_{m}$ less than 45 mV/ms were excluded. Note that the rapidity for RS and FS hippocampal neurons cross using the phase slope method.
