# Supplementary material for: New methods for quantifying rapidity of action potential onset differentiate neuron types
Source: PLoS One. 2021 Apr 8;16(4):e0247242. doi: 10.1371/journal.pone.0247242 (PMC8032118; doi:10.1371/journal.pone.0247242)
Supplement: S6 Fig — Blue circles show the mean phase slope value at different criterion levels for the cortical RS pyramidal neurons. Red squares show the mean phase slope value at different criterion levels for the cortical FS neurons. All APs that have maximum V˙m less than 45 mV/ms were excluded. (DOCX) [file pone.0247242.s006.docx]

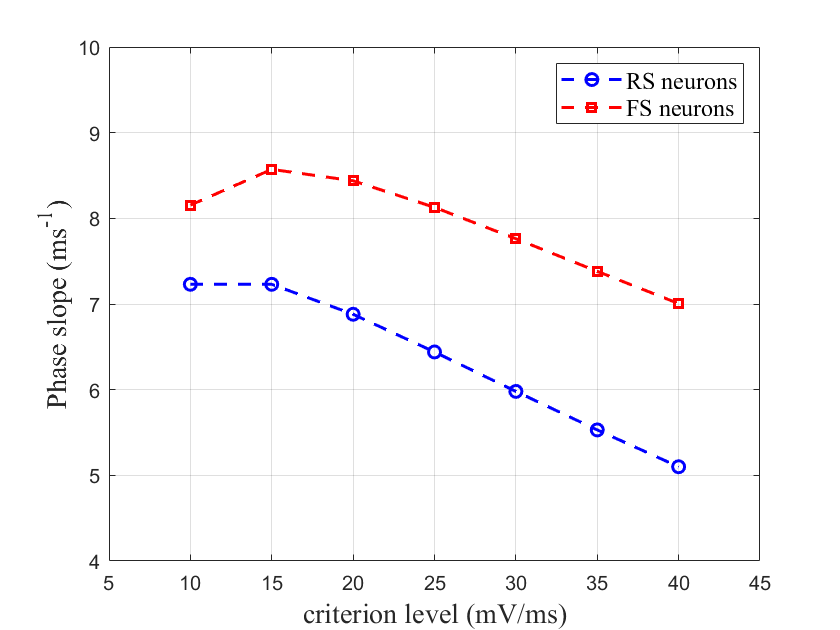


**S6 Fig. The impact of the onset criterion level on the phase slope for cortical neurons.** Blue circles show the mean phase slope value at different criterion levels for the cortical RS pyramidal neurons. Red squares show the mean phase slope value at different criterion levels for the cortical FS neurons. All APs that have maximum $\dot{V}_{m}$ less than 45 mV/ms were excluded.
