# Supplementary material for: New methods for quantifying rapidity of action potential onset differentiate neuron types
Source: PLoS One. 2021 Apr 8;16(4):e0247242. doi: 10.1371/journal.pone.0247242 (PMC8032118; doi:10.1371/journal.pone.0247242)
Supplement: S1 Table — (DOCX) [file pone.0247242.s007.docx]

**S1** **Table.** Electrophysiological properties using the pooled mean and standard deviation

|  | **Cortex** | | | | **Hippocampus** | | | |
| --- | --- | --- | --- | --- | --- | --- | --- | --- |
|  | **RS** | **FS** | **t** | **d** | **RS** | **FS** | **t** | **d** |
| **n** | 27 | 7 | ---- | ---- | 17 | 6 | ---- | ---- |
| **IFWd^2^**  **(ms^-1^)** | 2.1 ±0.3 | 2.7± 0.2 | 4.94^e*^ | 2.38 | 4.3 ±0.5 | 6.9 ±0.4 | 11.29^e*^ | 5.61 |
| **IHWd^2^**  **(ms^-1^)** | 4.0 ±0.5 | 4.6 ± 0.3 | 2.91^e*^ | 1.41 | 7.2 ±1.1 | 12.1 ±1.0 | 9.18^e*^ | 4.75 |
| **Phase Slope**  **(ms^-1^)** | 6.9 ±1.3 | 8.1 ± 1.1 | 2.27^e*^ | 1.00 | 47.8 ±64.6 | 10.4 ±4.5 | 2.37^*^ | 0.82 |
|  |  |  |  |  | 35.2±15.2^a^ | 11.1 ±4.6^a^ | 5.75^*^ | 2.15 |
| **Error ratio**  **(dimensionless)** | 7.9 ±6.4 | 11.2 ±7.5 | 1.85^e^ | 0.48 | 6.1 ±2.7 | 0.7 ±0.5 | 7.92^*^ | 2.79 |
|  |  |  |  |  | 8.5 ±2.5 ^b^ | 8.5 ±2.8^b^ | 0.00^e^ | 0.01 |
| **Amplitude**  **(mV)** | 61.7 ±8.6 | 58.0 ±5.2 | 1.06^e^ | 0.51 | 67.5 ±5.3 | 48.9 ±2.1 | 11.96^*^ | 4.58 |
| **Width**  **(ms)** | 2.0 ±0.3 | 0.8 ± 0.1 | 19.02^*^ | 5.59 | 1.6 ±0.4 | 0.3 ±0.01 | 13.81^*^ | 4.74 |
| **Onset potential**  **(mV)** | -26.3±6.0 | -39.5±3.3 | 5.56^e*^ | 2.74 | -30.2 ±3.5 | -33.4 ±1.1 | 3.30^*^ | 1.23 |

All data are expressed as mean ± SD. The RS neurons is pyramidal neuron, and the FS hippocampal neurons are PVBCS. ^a^using piecewise cubic interpolation. ^b^ the upper limit was set to 3 mV above the onset. ^*^ the difference is significant at p<0.05. d is the Cohen’s d effect size, and t is the t-score. For the t-score, ^e^ indicate that the equal variance hypothesis was accepted.
