# Supplementary material for: New methods for quantifying rapidity of action potential onset differentiate neuron types
Source: PLoS One. 2021 Apr 8;16(4):e0247242. doi: 10.1371/journal.pone.0247242 (PMC8032118; doi:10.1371/journal.pone.0247242)
Supplement: S2 Table — Green cells indicate a p-value below 0.05. (DOCX) [file pone.0247242.s008.docx]

**S2 Table.** Two-tailed p-value from the t-score between the IFWd^2^ values from different neuron types, and Cohen’s d effect size (in parentheses). Green cells indicate a p-value below 0.05.

| **AP width** | | **Cortex** | | **Hippocampus** | |
| --- | --- | --- | --- | --- | --- |
|  |  | **PCs** | **FS** | **PCs** | **PVBCs** |
| **Cortex** | **PCs** |  | <0.0001  (2.34) | <0.0001  (1.46) | <0.0001  (3.04) |
|  | **FS** | <0.0001  (2.34) |  | <0.0001  (3.08) | <0.0001  (4.09) |
| **Hippocampus** | **PCs** | <0.0001  (1.46) | <0.0001  (3.08) |  | <0.0001  (5.53) |
|  | **PVBCs** | <0.0001  (3.04) | <0.0001  (4.09) | <0.0001  (5.53) |  |
