# Supplementary material for: New methods for quantifying rapidity of action potential onset differentiate neuron types
Source: PLoS One. 2021 Apr 8;16(4):e0247242. doi: 10.1371/journal.pone.0247242 (PMC8032118; doi:10.1371/journal.pone.0247242)
Supplement: Attachment — Submitted filename: PLOS ONE rebuttal letter 6DEC2020Aldohbeyb.docx [file pone.0247242.s009.docx]

Dear Dr. Cymbalyuk,

We thank you and the reviewers for the comments on our manuscript, "New Methods for Quantifying Rapidity of Action Potential Onset Differentiate Neuron Types". We revised the paper to address the editor’s and the reviewers' comments and edited the manuscript accordingly. We believe the suggested additional statistical tests and discussion have made the paper stronger. On the following pages, you will find our response to the editor's and reviewers' comments.

On behalf of the co-authors, I thank you for considering our revised paper for publication.

Thanks

Ahmed Aldohbeyb

**The sample sizes that contain only 3 neurons of a particular type (CA1 hippocampal interneurons in Results section on page 17 – 18, Table 4 on page 21, the table is mislabeled as Table 1) are too small. It is not appropriate to use the t-test for such small groups because there is a substantial probability that all 3 measurements of the rapidity in these 3 neurons fall on one side of the distribution of the measure. Moreover, it is impossible to deduce what type of distribution is in case of such a small sample. Non-parametric statistical tests such as the Kruskal-Wallis test, which can be used also for a non-normal distribution, will show that such small groups are insufficient to make any conclusions. Therefore, either additional neurons should be included in these groups or all Results section describing differences between these groups and the corresponding Discussion section should be deleted.**

We excluded all neurons with a sample size equal to three. Such a decision was made after performing the Kruskal-Wallis test. The test showed that the combination of sample sizes between neurons with small and large sample size did not allow finding a critical value. Thus, we removed neuron types with a small sample size since the data were obtained from open-source databases, and we could not increase the sample size.

A non-parametric test was performed to evaluate the different AP rapidity quantification methods. The non-parametric Mann-Whitney U test was performed to compare different cell types using each of the rapidity quantification methods. This test was used in many of the classical classification papers such as McCormick et al. (1985) and [Nowak et al. (2003](https://www.sciencedirect.com/science/article/pii/S0896627310000590#bbib43)). The results from the Mann-Whitney U test showed that all the rapidity quantification methods can differentiate between neuron types. Nonetheless, the IFWd^2^ method still had the highest effect size, t-score, and z-score (Table 3).

**Use of pooled mean: In the case of statistics by type, the use of a pooled mean weighted by number of spikes seems a bit odd, since the primary source of variation is likely to be at the cell level, not the spike level. The pooled SD is therefore certainly not a good estimate of the variability of the population mean, and the population mean itself will be biased towards cells with more spikes. Finally, it is unclear if these pooled calculations are also used in the t-tests, which would certainly overstate the differences between types. T-tests: the authors should justify whether the equal variance assumption of Student's t-test is met here, or use a different test.**

We have performed conventional statistical analysis, where the APs from all cells of a given type were combined into a single sample group and analyzed and then use these conventional means and SDs in the statistical comparison of rapidity quantification methods. We had initially chosen the pooled statistics because we believed cells within a specific cell type taken from the same region should have similar electrophysiological properties. However, the reviewer raised a valid argument. So, we took the first 50 APs from each cell and calculated the mean and SD of all the APs obtained from a specific cell type. The t-score and z-score was calculated for the conventional statistics (Table 3) and the t-score for pooled statistics was moved to supplemental material (Table S1). Also, in these tables, we indicate if the equal variance assumption was accepted, and use the Welch t test when the equal variance hypothesis was rejected.

**Classification performance would be directly related to the effect size of the test, which is not discussed, and ideally be demonstrated directly by training a classifier and then evaluating performance on untrained test data. The authors should at a minimum add calculations of effect size in terms of Cohen's d, common language effect size, or both.**

As recommended, effect size test was performed to compare the different AP rapidity quantification methods. Cohen's d effect size test was performed on both conventional (overall) and pooled (effectively two-tier) mean and SD. The Cohen’s d effect size are included in Table 3.

**Action potential backpropagation and, more precisely, the dendritic shunting, mentioned in the Yu, Shu and MacCormick paper of 2008 and investigated in detail by Eyal, Mansvelder, Kock and Segev (2014), are, probably, more important factors determining the rapidity of the action potential onset. Therefore, both the introduction and, especially, the discussion should be modified to reflect this fact. In addition, the last sentence in the Abstract should be modified accordingly.**

**For the comparison of hippocampal to cortical cells, the authors should address whether factors in addition to intrinsic differences could be contributing to the cross-type distinctions shown. Specifically, recording conditions likely differed between the two data sources, and should be discussed.**

A detailed discussion was added to address the factors that could cause the difference in AP onset rapidity between neuron types (line 455-495 in the unmarked manuscript). In the new section in the discussion headed “Factors affecting AP onset rapidity”), we discussed the different mechanisms suggested in the literature to explain the difference in AP rapidity such as AP backpropagation, somatodendritic size, and cooperative gating. Also, we acknowledge that some of the differences between cortical and hippocampal neurons could be due to differences in recording and preparation conditions (line 507-515). Finally, we would like to note that the focus of the paper is on the robustness of the proposed methods for AP rapidity analysis.

**Implications for underlying mechanisms: "Contrary to published reports on the maximum rise rate, the difference in AP rapidity between neuron types indicates a significant difference in the activation kinetics of voltage-gated sodium channels (VGSCs)." (l38) This statement is not well supported – elsewhere in the manuscript the authors mention the significant discussion in the literature over whether non-channel factors (passive interactions between compartments) contribute to differences in onset. They should therefore also acknowledge that these factors could provide alternative explanations for cross-type differences. (Input resistance differences are mentioned briefly as a possible factor on l473, but not elsewhere). In addition, when discussing how their results fit with the existing literature, it would be helpful for the authors to distinguish between the backpropagation [7] and critical resistive coupling [9, 14] theories, which are both alternative explanations for variations in AP onset not linked to sodium channel properties**

As recommended, we added a detailed discussion and acknowledge the different factors that could contribute to the difference in AP rapidity (lines 455-495). Also, we added the result comparing single and double-peaks in the second derivative trace, which is a characteristic for AP backpropagation (lines 390- 411 and 460-475). Also, we deleted the statement ("Contrary to published reports…") from the abstract.

**When discussing the impacts of the interpolation, the following statement is not sufficiently clear: “****significant reduction in the phase slope values is expected since the slope is calculated at a specific value (at 10 mV/ms) of the dependent (vertical axis) variable in phase space plots.”**

In the revised manuscript we clarify the sentence with additional discussion on line 250-256. Unlike the spline interpolation, the cubic piecewise interpolation does not produce an excursion between the recording. Thus, instead of producing a bowed excursion between two data points, the cubic piecewise interpolation, never produces an excursion beyond the data points between which it is interpolating, and hence decreased the variation in the phase slope values.

**On lines 65 – 67 references are cited inappropriately. Reference 8 does not talk about 'cooperative gating': in this paper a simple 6 state model without any cooperation fits well sodium current traces that were found to be without a HH-type delay. In reference 7 backpropagation of action potentials alone is considered to be sufficient to account for the experimental data and the 'cooperative' gating model was found to be unnecessary. Please modify this sentence accordingly.**

We apologize for the misplaced references in our submitted manuscript, which the editor noted. The reference numbering has been corrected (line 64-68).

**The numbering of tables is confused in several places:
- in p. 3 probably should be referred to the Table 1 but not Table 3;
- Table 1 is probably Table 4 (p. 21), this table is referred as Table 1 on page 20.**

We apologize for this numbering mistake as well, which has now been corrected. The tables were rearranged as the editor suggested. Table 1 was moved to page 9, and Table 2 was deleted since many hippocampal neuron types were deleted from the manuscript due to small sample sizes and the revised Table 1 summarizes the results from all the remaining 4 neuron types. Also, we added a new Table 3, which summarize the results from the different statistical tests.
