# Supplementary material for: New methods for quantifying rapidity of action potential onset differentiate neuron types
Source: PLoS One. 2021 Apr 8;16(4):e0247242. doi: 10.1371/journal.pone.0247242 (PMC8032118; doi:10.1371/journal.pone.0247242)
Supplement: Attachment — Submitted filename: response letter 12Jan2021.docx [file pone.0247242.s010.docx]

Dear Dr. Cymbalyuk,

We thank you and the reviewers for the comments on our manuscript, "New Methods for Quantifying Rapidity of Action potential Onset Differentiate Neuron Types". We revised the paper to address the editor and the reviewers' comments and edited the manuscript accordingly. As with the earlier revision, we believe your and the reviewers’ suggestions have made the paper stronger. On the following pages, you will find our response to specific comments.

On behalf of the co-authors, I thank you for considering our revised paper for resubmission.

Thanks

Ahmed Aldohbeyb

The manuscript should be revised for readability.

As recommended, the manuscript was reviewed and revised to enhance readability by the three authors as well as an outside technical writer and editor, Sierra Lear, who is the newly appointed editor-in-chief for the Berkeley Science Review.

Abbreviations in tables should be used more consistently: Somatosensory cortex in Table 1, S.S. Cortex in Table 3 and SS cortex in Table 4.

We apologize for the inconsistent abbreviations in the table. As recommended, all tables have been updated to have the same abbreviations.

It could be useful to have a list of abbreviations, some of them are difficult to interpret and may be confusing, for instance VGKC and VGSC.

As recommended, a list of abbreviations is included in the manuscript (lines 42-46). We would be happy to revise the formatting to any style guide or other guidance during the proof stage if the paper is accepted.

On line 332 'methods have the highest values on all three statistics' probably should read 'methods have the highest power according to all statistical tests used'.

The sentence was modified to “have the highest scores of all methods in Table 3 according to all three statistics” since the power was not reported in Table 3.

The new analysis comparing single and double-component could be shortened and made more readable by focusing on a single metric, probably IFWD, and presenting the details of which comparisons are significant in a table.

The text describing comparative analysis of AP waveform components was considerably shortened, and a new table was added to summarize the results (Table 4), as recommended.

The common language effect size for the Mann-Whitney test (equivalent to the area-under-curve from ROC analysis) might be a more relevant effect size for situations in which the non-parametric MW test is considered the primary test indicated by the properties of the data.

As recommended, the effect size was measured using the common language effect size test, as well as Cohen's d test. The results are shown in Tables 3 and 4.

reviewer #1: Although the authors adequately addressed all issues raised in my comments there are still language problems in the text that should be addressed. First, abbreviations in tables should be used more consistently: Somatosensory cortex in Table 1, S.S. Cortex in Table 3 and SS cortex in Table 4. Second, on line 332 'methods have the highest values on all three statistics' probably should read 'methods have the highest power according to all statistical tests used'. Third, it could be useful to have a list of abbreviations, some of them are difficult to interpret and may be confusing, for instance VGKC and VGSC. Finally, the manuscript should be made more readable by somebody with more experience in paper writing of by professional editing services, there are many places that are difficult to follow.

We thank the reviewer for their insightful comments. We apologize for the inconsistent abbreviations in the table. We fixed these issues and added a list of abbreviations on the first page. Also, as suggested, the sentence in line 332 was modified, and the manuscript was revised for readability and was then reviewed and revised by the editor-in-chief for the Berkeley Science Review.

Reviewer #2: The authors' revisions are appreciated, and have substantially increased the quality of the manuscript. I support publication in the current form, but have a few minor, non-essential edits to suggest.
The addition of the "Factors affecting AP onset rapidity" section in the discussion is very useful. It could be additionally improved by minor edits noting which developments are supported by computational modeling vs experimental evidence. Likewise, the new analysis comparing single and double-component APs adds significantly to the links to this prior work in the field. It could, however, be shortened and made more readable by focusing on a single metric, probably IFWD, and presenting the details of which comparisons are significant in a table. Finally, although I think the broad presentation of alternative statistics in the current version is more than sufficient, I'd like to note that the common language effect size for the Mann-Whitney test (equivalent to the area-under-curve from ROC analysis) might be a more relevant effect size for situations in which the non-parametric MW test is considered the primary test indicated by the properties of the data.

We thank the reviewer for their insightful comments. The discussion section on factors affecting AP onset rapidity was updated to indicate the type of evidence used in the cited studies. For the analysis comparing AP waveforms with single and double components, we shortened the section and summarized the results in a table instead, as suggested. Finally, we used the common language effect size test in all comparisons as shown in Tables 3 and 4.
